#  Fungal keratitis: study of increasing trend and common determinants 

**DOI:** 10.3126/nje.v7i2.17975

**Published:** 2017-06-30

**Authors:** Yogesh Acharya, Bhawana Acharya, Priyanka Karki

**Affiliations:** 1 Assistant professor, Avalon University School of Medicine, Willemstad Curacao, Netherland Antilles.; 2 Registered nurse, VHA home health care, Toronto Ontario, Canada.; 3 Medical officer, Nobel Medical College and Hospital, Biratnagar Morang, Nepal.

**Keywords:** Fungal eye Infections, contact lenses, risk factor, prevalence, complication

## Abstract

Fungal keratitis is one of the leading cause of ocular morbidity. Fungal keratitis possesses a clinical challenge due to its slow pathologic process, overlapping features, diagnostic difficulty, and potential complications. Its increasing trend can be attributed to the use of contact lens, non-judiciary corticosteroid, and vegetative trauma. Early diagnosis and treatment is the cornerstone for its effective control. Knowledge of pathological course and clinical characteristics of fungal keratitis will definitely add in early diagnosis and treatment, with reduction in ocular morbidity. This review article explores the risk factor of fungal keratitis, its clinical course and management strategy.

## Introduction

Eye is the most beautiful thing in the world. Pure appreciation of beauty is only possible with the intact functioning eye. It is well said that vision controls the mind. We believe in what we see and we prefer to see what we believe in. Eye is a delicate organ and is kept free of pathogens and harmful microorganism by its natural protective mechanism. This natural check and balance is absolutely necessary for a healthy eye. Breach of this delicate balance of protective environment can lead to ocular diseases with visual morbidity. 

 Cornea is the major refractive and protective outer layer of eye. Inflammation of the cornea is known as keratitis. There are several causes of keratitis: infectious, physical or chemical. Infectious or microbial keratitis is a predictor of general health due to its higher incidence in community and associated complications. Microbial keratitis has long been a challenge for the physicians ' due to its varied presentation, overlapping symptoms, and rapid progression. Bacterial keratitis is the most prevalent amongst microbial keratitis. But there has been a constant surge in fungal keratitis in the recent times due to multiple overlaying factors. Despite being a slow process in comparison to bacterial counterpart fungal keratitis possesses considerable ocular morbidity. Fungal keratitis carries a significant risk in developing countries and is one of the leading causes of vision loss [ 1 ]. Vegetative trauma in agriculturist and sand particles are the most common causes of mycotic keratitis in developing countries [ 2 ]. Middle age workingmen are more susceptible and constitutes majority of cases. Whereas, contact lens (CL) use is the leading cause in developed countries. 

 Fungi are distinct group of microbial organism. They are ubiquitous microbial eukaryotic pathogens. Fungal keratitis, also known as keratomycosis, is an important cause of microbial keratitis in the general population. Fungi are classified as yeasts or moulds. Yeasts ore oval or round bodies, with blastoconidium. Moulds are filamentous structures, known as hyphae. Hyphae can form a large mass of filaments known as mycelium. These filaments can be septate or non-septate. Fungi are capable of reproducing sexually as well as asexually. Sexual reproduction takes place through formation of spores and asexual by conidia or sporangiospores. Fungi infecting cornea are generally in asexual phase of life cycle, when cultured. Yeast like fungi are associated with the past history of ocular diseases, surgery and steroid use with poor clinical outcome. Filamentous fungi are usually found in patients with a history of ocular trauma [ 3 ].

### Epidemiology:

There are more than 900,000 physician visit and 58,000 emergency visits related to keratitis and contact lens use in US, accounting an estimated expenditure of around $175 million dollar in health care [ 4 ]. Contact lens provides a direct threat for microbial keratitis and around 26 million contact lens users have significant ocular health risk. Fungal keratitis is also an important predictor of ocular health in developing countries and is a major cause of unilateral blindness

### Risk factors:

Eye is susceptible to microbial infection due to local and systemic factors that invade the protective mechanism. These local factors like trauma to the eye ball, introduces and inoculates various fungal members into the eye with or without bacterial associates. Risk factors associated with fungal keratitis are Male, Trauma, Contact lens use, Topical corticosteroid use, Diabetes mellitus, and Low socioeconomic status.

Vegetative ocular trauma is undoubtedly the most common risk factor for fungal keratitis. Ocular trauma is essential to breach an intact corneal epithelium for introduction of microbial organism. Vegetative trauma predisposes to fungal infections being an important identifiable cause. It is extremely rare to encounter a case of fungal keratitis in an otherwise healthy eye, without any associated risk factors. This is because intact cornea is fairly resistant to microbial infections. Trauma helps to introduce and inoculate fungi directly into the cornea. Male are particularly more prone to fungal keratitis, as outdoor activities and farming practices predisposes to vegetative trauma.

CL use has become widespread in recent times. With an increase in CL use in the general community, the overall cases of fungal keratitis are also increasing. Although CL is implicated for major proportion of fungal keratitis, the overall prognosis is better in contact lens induced keratitis [ 3 ]. Factors associated with CL use and chances of fungal keratitis include nocturnal use during sleep, male gender, smoking history and socioeconomic status, relating to unhygienic contact lens behavior [ 5 ]. Microbes have higher chance of adherence to cornea with CL. Hypoxia and hypercapnia are pathogenic changes associated with CL induced microbial keratitis [ 6 ]. 

 Antibiotics and corticosteroids use also render the eye susceptible to infections. Steroid has been the cornerstone of medical management for inflammatory disease process in modern medicine. Excessive steroid use leads to decrease in host defense mechanism and creates a favorable environment for fungal inoculation. Systemic disease like diabetes mellitus has emerged as a major risk factor in the recent years. Diabetes is becoming a global public health problem. Host defense is severely impaired in diabetes and high glucose provides a suitable growth media for microbial organism.

### Fungal species:

*Apergillus* and *Fusarium* are two major cause of fungal keratitis.* Aspergillus*is associated with higher incidences of complications but shows better response with antifungal medications. Fungal keratitis due to *Candida * species has the worst clinical outcome [ 7 ]. Common fungal isolates from corneal scrapping of patients with clinically suspected keratitis are listed in Table-2.

*Fusarium * is mainly associated with contact lens induced keratitis. It is necessary to recognize *Fusarium * keratitis early in the period of its progression and adequate measures should be taken to minimize the ocular morbidity [ 8 ]. There have been multiple reported incidences of *Fusarium * keratitis with CL and ReNu CL solution (Bausch & Lomb) for lens care. This can be attributed to poor contact lens hygiene and improper use habits. Some of these patients required emergency penetrating keratoplasty for severe complicating keratitis despite the medical treatment [ 9 ]. There are different *Fusarium * species and complexes responsible for keratitis; *Fusarium solani* species complex, *Fusarium oxysporum* species complex and *Gibberella fujikuroi* species complex. *Fusarium * solani species complex is the most common [ 10 ]. 

 There are many other fungal species implicated in fungal keratitis. These fungal species are responsible for sporadic cases of fungal keratitis and include: *Lophotrichus* spp. [ 11 ], *Alternaria* spp. [ 12 ], *Acremonium* spp. [ 13 ],* Cryptococcus albidus *[ 14 ]),* Pythium insidiosum* [ 15 ],* Beauveria bassiana* [ 16 ],* Paecilomyces * [ 17 ], *Cunninghamella spinosum* [ 18 ], *Scedosporium apiospermum* [ 19 ]), *Rhodotorula mucilaginosa* [ 20 ],* Cylindrocarpon lichenicola* [ 21 ], *Cladorrhinum bulbillosum *[ 22 ].* Lophotrichus* species were isolated from the necrotic corneal sample complicating fungal keratitis after dog paw traumatic injury [ 11 ].

### Presentation:

Keratitis usually presents with ocular pain, foreign body sensation and blurred vision. Affected eye will be red and the patient can have injection of the conjunctiva. Intense inflammatory process after infection usually results in copious amount of ocular secretions but secretions in fugal keratitis are usually scanty in contrast to other microbial infections.

### Diagnosis:

Diagnosis of fungal keratitis starts with strong clinical suspicion with concurrent presence of risk factors. Diagnosis is strongly supported by suggestive clinical presentations and fungal isolation from corneal sample. Antibiotic unresponsiveness provides a clinical clue for diagnosis. Direct microscopic examination of cornea with subsequent of corneal sample culture, still remains the gold standard for fungal diagnosis.

**Fig-1:**
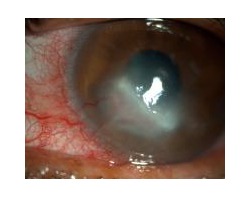
Peripheral corneal fungal keratitis in slit-lamp microscopy.

**Fig-2:**
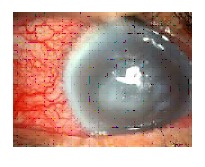
Fungal keratitis with satellite lesions in slit-lamp microscopy.

**Fig-3:**
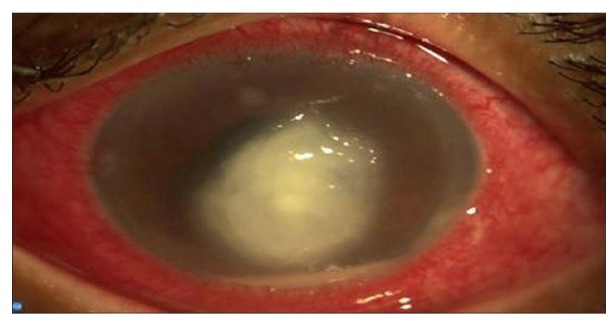
Corneal fungal ulcer with Hypopyon in slit-lamp microscopy.

Keratitis is best examined under slit lamp microscopy. Slit lamp microscopy will show dry, thick and raised corneal surface. Majority of cases will have stromal infiltrates with feathery margins and endothelial plaques (Fig-1).Satellite lesion is typically seen in fungal keratitis (Fig-2). Hypopyon is detected in most of the cases, which can lead to ocular hypertension (Fig-3). The identified risk factor for hypopyon includes infection with *Fusarium and Aspergillus*, in particular; and long duration of symptoms with larger lesion size [ 23 ]. It is not uncommon to see deep stromal infiltration, corneal abscess and dissemination of infection.

Once the presumptive diagnosis is made, corneal scrapping and corneal biopsy is taken as required for inoculation and isolation of organism. There are different ways to illustrate the presence of fungal keratitis. These methods include Gram’s stain, Potassium hydroxide (KOH) mount, and Calcofluor white fluorescent staining and finally culture. Common culture media for fungus is Sabourauds Dextrose Agar. Direct visualization with KOH wet mount is commonly implicated followed by culture, being the most conclusive. (2) KOH is applied in corneal scrapping to dissolve epithelial strands. Use of 10% KOH better aids in fungal recognition and diagnosis. Definitive diagnosis lays on the foundation of culture and isolation. Culture facilitates the direct visualisation of fungi under microscopy. Culture and sensitivity will also guide the use of appropriate anti-fungal therapy for superior clinical recovery.

Polymerase Chain Reaction [ 24 ] and dot hybridization [ 25 ] are newer rapid detection technique for sensitive and specific diagnosis of fungal species. They have higher sensitivity than KOH wet mount and Gram smear [ 26 ] and are able to detect the fungus successfully in culture negative cases [ 27 ]. PCR high-resolution melting analysis is a variant PCR technique that is effective in differentiating between filamentous fungi and yeast form [ 28 ].

### Management:

Management of fungal keratitis is directed by the objectives: a) distinctive diagnostic procedure to correctly identify the disease process, b) concurrent use of effective treatment modalities, c) eradication of the disease process, d) minimizing the complication, and e) prevention of future recurrences. This is achieved by the use of topical antifungal medication with or without surgical interventions. It is absolutely necessary to halt the disease progression early in the pathogenic process to reduce the overall complications and associated ocular morbidity.

### Pharmacological management:

Pharmacological treatment of fungal keratitis rest on topical anti-fungal medications. There are currently no available antifungal recommendations in accordance with specific fungal isolation. Many of these anti-fugal differ in their corneal penetration activity and effectiveness. Topical instillation of the active antimicrobial pharmacological agents is still remains the gold standard treatment protocol. There are conflicting reports of intra-stromal injections but it has not shown proven added benefit over topical instillation [ 29 ].

 Natamycin remains the cornerstone of anti-fungal therapy. Natamycin (5%) is the treatment of choice for filamentous fungi. Poor response to natamycin is directly related to increase infiltrate, larger scar size and perforation probability [ 30 ]. For candida species topical amphotericin B (0.1-0.3%) is frequently implicated with superior response [ 31 ]. There has been increasing evidence of topical voriconazole use in fungal keratitis with favourable clinical outcome. Topical voriconazole is especially useful in fungal keratitis not responding to natamycin [ 29 ]. Topical voriconazole is also effective against *Cladosporium* species [ 32 ]. There are different patterns of drug susceptibility in different groups of fungi. Therefore it is warranted that fungal specimen is taken and culture grown, for identification and antimicrobial sensitivity, if no response is visible after initiation of treatment [ 33 ].

Some fungal infection responds to topical fluoroquinolones, namely moxifloxacin [ 34 ]. This initial response leads to assumption of bacterial infection and eventually accounts for higher chances of complications with delay in diagnosis. It is necessary to understand that fluoroquinolone monotherapy will not be able to control most of the fungal infection and in turn can lead to prolongation of the disease course with chances of relapse.

### Surgical management:

Surgery is definitely a choice for fungal keratitis, when response to pharmacological agent is poor and there is imminent threat of perforation. Surgery will eliminate the necrotic, infectious and antigenic source of ocular insult with creation of favourable environment for pharmacological agents to act and fastens healing [ 35 ]. 

 Periodic debridement is an excellent procedure to remove dead and necrotic tissues from cornea. Debridement helps to improve the blood circulation, increase topical drug effectiveness and finally decrease the overall microbial load for speedy recovery. Conjunctival flap and lamellar or penetrating keratoplasty is applied in severe keratitis where a pharmacological agent fails. Patch graft and transplant can be used as final resort to restore the cornea and normal vision, whenever possible.

### Corneal crosslinking:

Corneal crosslinking is an effective approach to control microbial keratitis. It is proven to have an excellent ulcer healing properties and induce overall reduction in hypopyon formation. There are few incidences of opacification of lens after crosslinking procedure [ 36 ]. Its efficacy is limited in viral keratitis due to incidences of corneal melting and tectonic keratoplasty [ 37 ]. Corneal collagen cross-linking with photoactivated riboflavin (CXL-PACK) has shown a promising outcome in-patient with advanced microbial keratitis with corneal melting. They decrease corneal perforation and recurrences in majority of patient [ 38 ]. CXL-PACK is safer procedure but it can increase the likelihood of bacterial keratitis in the patient because of epithelial removal necessary for the procedure. In addition to to it there are other potential factors responsible for causing keratitis, which can range from use of contact lens after local corticosteroid after the procedure. It is necessary for the physician to properly counsel the patient about this complication and caution should be taken to avoid them [ 39, 40 ].

### Complications:

**Fig-4:**
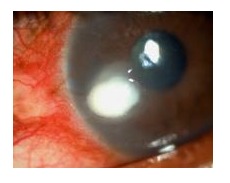
Corneal fungal abscess at periphery with infiltration in slit-lamp microscopy.

Fungal keratitis can result in several complications leading to visual disability. These complications can range from formation of abscess and mild to severe corneal scarring with loss of vision due to dissemination (Fig-4). Severe long-term disease process can lead to corneal perforation and dissemination of infection. Fungal infection also facilitates other superadded microbial keratitis. Patient can have an anterior segment disruption with increased intraocular pressure leading to glaucoma. Similarly it can result in endophthalmitis resulting in evisceration making the patient visually handicap.

### Recent developments:

There are newer approaches for diagnosis of fungal corneal infection and treatment methods. Dot hybridization assay has been used successfully to diagnose *Cryptococcus albidus*, a rare fungi which and treated with intra-stromal injection of Amphotericin B [ 14 ]. MicroRNAs (MiRNAs) are RNA molecules in humans that do not code. They are responsible for regulation of functions in the human cell and show very high tissue specificity. They can be identified by PCR technique. They are evidences of increased MiRNA expression in fungal keratitis, indicating its role in pathologic process and potential target for modifications in the future [ 41 ]. 

 Calprotectin is a neutrophil derived protein with potent antimicrobial property. Calprotectin uses Zn and Mn chelation to inhibit fungal growth. It has proven beneficial effect in *Aspergillus fumigatus* growth inhibition in experimental mice model and awaits further work in humans [ 42 ]. Lectin-like oxidized low-density lipoprotein receptor 1 (LOX-1) [ 43 ], spleen-tyrosine kinase (Syk) [ 44, 45 ] and intracellular nucleotide-binding oligomerization domain-containing protein (NOD)-like receptors [ 46 ] have also been isolated from *Aspergillus* infected corneal epithelial cells. LOX-1, Syk and NOD have basic role in modification of signalling pathways and its inhibitors can be used to regulate fungal growth in the future. 

 Photodynamic therapy with rose Bengal has been successful in restriction of certain fungal growth [ 47 ]. Riboflavin/UV-A has also been effective in *Fusarium, Aspergillus *and *Candida* after pre treatment with amphotericin B [ 48 ]. Combination therapy of antifungal medication with UV-A can prove safe and alternative to single therapy [ 49 ].

## Discussion

There has been progressive incline in microbial keratitis in the recent time. Fungal keratitis, in particular, has the highest risk and possesses a significant threat for increased ocular morbidity owing to its slower course and diagnostic difficulty. This rapid surge can be attributed to increased contact lens use, and non judiciary antimicrobial and corticosteroid use, complicated by systemic disease interfering with immune status. Most of the cases in western world can be attributed to unhygienic contact lens use, whereas vegetative trauma in working class is the major cause in developing countries. Male has higher incidences of fungal keratitis but corneal re-epithelialization time is higher in female in comparison with males, accounting higher recovery period [ 50 ]. 

*Fusarium* and *Aspergillus* are the common isolates from corneal scrapping, *Fusarium* associated with CL use. Healthy eye is relatively immune to fungal infection and ocular trauma provides an excellent opportunity for pathologic fungal inoculation into eye. Fungal keratitis has a relatively slow progression with dreadful complication that can range from corneal scarring to perforation and eventually loss of vision. Presence of satellite lesion is a strong indicator of fungal origin and strong clue can be provided by the fact that fungal corneal ulcers are not responsive to traditional antibiotics. 

 There is a major reduction in ocular morbidity with early diagnosis and treatment [ 1 ]. The traditional approach consists of slit lamp microscopic examination and corneal scrapping for identification and culture. There are newer rapid diagnostic technique with higher sensitivity and specificity like PCR. The prompt diagnosis is to be followed by effective management. Management is guided by topical antifungal therapy and surgery, if needed.

## Conclusion

Fungal keratitis is one of the leading causes of ocular morbidity. Recognized common risk factors include vegetative trauma, widespread contact lens use, prolonged non-judiciary corticosteroid prescription, and systemic disease like diabetes mellitus. Fungal keratitis possesses a clinical challenge due to its slow pathologic disease process, overlapping features with other microbial keratitis and potential complications. The knowledge of clinical characteristics of fungal keratitis with its determinants will certainly help in early diagnosis and overall reduction in visual morbidity associated with it.

## List of abbreviations:

1. Contact Lens (CL)
2. Potassium Hydroxide(KOH)
3. Polymerase Chain Reaction(PCR)
4. Collagen cross-linking with photoactivated riboflavin (CXL-PACK)
5. MicroRNAs (MiRNAs)
6. Lectin-like oxidized receptor-1 (LOX -1)
7. Spleen-tyrosine kinase (Syk)
8. Nucleotide-binding oligomerization domain (NOD)

## Figures and Tables

**Table-2:** Commonly isolated fungi in microbial keratitis.

Fungi	
Common	Uncommon
1. Fusarium	1. Lophotrichusspp.
2. Aspergillus	2. Alternaria spp.
3. Candida	3. Acremonium spp.
4. Cladosporium	4. Cryptococcus albidus
5. Curvularia	5. Pythium insidiosum
6. Rhizopus	6. Other :Beauveriabassiana, Paecilomyces, Cunninghamella spinosum, Scedosporiumapiospermum, Rhodotorulamucilaginosa, Cylindrocarponlichenicola, Cladorrhinumbulbillosum.
